# Editorial: Childhood vaccination and COVID-19

**DOI:** 10.3389/fped.2023.1298691

**Published:** 2023-11-21

**Authors:** Tauqeer Hussain Mallhi, Muhammad Salman, Yusra Habib Khan, Faiz Ullah Khan, Muhammad Hammad Butt, Carolina Oi Lam Ung, Amjad Khan, Raja Ahsan Aftab

**Affiliations:** ^1^ Department of Clinical Pharmacy, College of Pharmacy, Jouf University, Sakaka, Saudi Arabia; ^2^ Institute of Pharmacy, Faculty of Pharmaceutical and Allied Health Sciences, Lahore College for Women University, Lahore, Pakistan; ^3^ Department of Pharmacy Administration and Clinical Pharmacy, School of Pharmacy, Xi’an Jiaotong University, Xi’an, China; ^4^ Department of Medicinal Chemistry, Faculty of Pharmacy, Uppsala University, Uppsala, Sweden; ^5^ State Key Laboratory of Quality Research in Chinese Medicine, Institute of Chinese Medical Sciences, University of Macau, Taipa, Macao SAR, China; ^6^ Department of Pharmacy, Quaid-i-Azam University, Islamabad, Pakistan; ^7^ Department of Clinical Pharmacy and Practice, Faculty of Pharmacy, Universiti Malaya, Kuala Lumpur, Malaysia

**Keywords:** COVID-19, childhood vaccination, vaccine hesistancy, conspiracy theories against vaccines, vaccination campaigns, parents’ perception towards vaccine

**Editorial on the Research Topic** Childhood vaccination and COVID-19

The 21st century will be indelibly marked by the global pandemic of Coronavirus disease 2019 (COVID-19), which has claimed approximately 7 million lives (1). The first case of COVID-19 was reported in December 2019 in Wuhan, China, and its causative agent severe acute respiratory syndrome coronavirus 2 (SARS CoV-2) was identified in January 2020. The World Health Organization (WHO) initially referred to the disease as 2019-nCoV and the term COVID-19 was coined in February 2020. On March 11, 2020, after more than 118,000 cases in 114 countries and 4,291 deaths, the WHO declared COVID-19 a pandemic (2). Pandemic management consisted primarily of preventative measures, drug repurposing, and vaccination. In December 2020, the vaccination program was expanded to include Pfizer and Moderna vaccines as a result of Emergency Use Authorization (EUA). However, these vaccines were initially recommended for adults and those at high risk. The FDA extended the use of Pfizer-BioNTech to adolescents aged 12–15 years in May of 2021 due to the vaccine’s adequate coverage of the adult population and balanced supply. Vaccinations were eventually recommended for children of all ages (3).

During the pandemic, vaccination campaigns against COVID-19 encountered numerous challenges. Vaccine hesitancy (VH) fueled by conspiracy theories against vaccines (4), a shortage of vaccines in certain areas, a lack of personnel at vaccination centers, and vaccination-related side effects were common barriers during COVID-19 vaccination programs (5, 6). These obstacles significantly impacted the COVID-19 vaccination measures on multiple fronts. VH during the COVID-19 pandemic was a profound phenomenon that became more severe after the introduction of COVID-19 vaccines for children. Successful vaccination campaigns rely primarily on achieving the highest possible coverage rate, which cannot be achieved without vaccinating children. VH against COVID-19 among parents drew the attention of healthcare professionals and policymakers at the beginning of 2022. Understanding parents’ perception toward childhood vaccination, the real-world estimation of side effects of COVID-19 vaccines among children, and the barriers to vaccination programs is crucial for developing and implementing targeted policies. In this context, we started a research topic (Childhood Vaccination and COVID-19) to evaluate various factors affecting the childhood COVID-19 vaccines.

Determining the factors associated with parental VH will help health officials develop educational campaigns. Alenezi et al. conducted a cross-sectional survey in Saudi Arabia to determine the relationship between psychological antecedents and parental willingness to vaccinate their children by using a validated scale named “5C” which measures five psychological factors (collective responsibility, complacency, confidence, calculation, and constraints). This study found that 54% of parents of children aged 5–11 years were unwilling to vaccinate their children, while 57.2% of parents of children aged 12–18 years were unwilling to administer booster doses. Of five psychological antecedents, confidence was associated with willingness and positively correlated with collective responsibility. However, complacency was associated with unwillingness to vaccinate children aged 12–18 years. A similar kind of study was conducted by Choi et al. in Macao, where the authors reported positive intention for childhood COVID-19 vaccination in less than half (42.5%) of the study population. Safety concerns were the major reasons for not having the intention to vaccinate their children. Low et al. also conducted a study among 628 Singaporean parents, reporting that 27.2% of parents were hesitant towards childhood vaccination. This study found that being a father, having a low household income, having unvaccinated parents, knowing someone who had an adverse reaction to the COVID-19 vaccine, and having a low level of trust in pediatricians were all associated with parental vaccination hesitancy.

Fakonti et al. investigated mothers’ attitudes and perceptions about COVID-19 vaccination for their children in Greece. This study included 1,885 mothers, and most of them (91.7%) had strong beliefs about the usefulness of COVID-19 vaccines. This study also demonstrated that previous maternal vaccination practices were associated with a positive attitude toward childhood COVID-19 vaccination. Khan et al. conducted a systematic review of 108 studies to determine the barriers and facilitators of childhood COVID-19 vaccination. They found the highest VH (approximately 80%) among people from the United States of America, Saudi Arabia, and Turkiye. This review indicated lower mothers’ education, financial instability, low confidence in new vaccines, and unmonitored social media platforms as commonly reported barriers to childhood COVID-19 vaccination. On the other hand, the study found that higher education levels, recommendations from healthcare professionals, and strong confidence in preventive measures taken by the government were found to be the facilitators of vaccine uptake.

The importance of pediatricians` recommendations for childhood COVID-19 vaccines has been well discussed in the literature. Mondal and Sinharoy conducted a national survey in the United States of America. They found that pediatricians’ positive recommendation is significantly associated with parental acceptance of the COVID-19 vaccine for their children. We believe that the participation of pediatricians in vaccination campaigns will assist health authorities in increasing vaccine uptake by reducing parental concerns regarding the safety of vaccines, particularly among parents of younger children.

Existing literature also discusses various factors associated with the uptake of the COVID-19 vaccine among children during the pandemic. Hoshen et al. investigated the determinants of vaccination compliance among children. The authors reviewed 61,776 children registered in their clinic and found a higher vaccination rate among children aged 9–11 years than children aged 5–6. The multivariate analysis indicated five independent determinants to be linked with low vaccination rates, including Arab and ultra-orthodox, children aged 5–8 years, children of low socioeconomic status, and children who had not received previous seasonal influenza vaccination. On the other hand, the vaccination rates were higher among children with comorbid conditions. These findings indicate a need to investigate further socio-demographic factors associated with vaccination compliance among children.

Despite the fact that pediatric COVID-19 vaccines have demonstrated promising safety in clinical trials and real-world pharmacovigilance studies, parents remain concerned about safety due to a few reports of multisystem inflammatory syndrome (MIS) and myocarditis following COVID-19 vaccination (7). Saeed et al. reported a case of an 11-year-old female who presented with MIS, possibly due to inactivated COVID-19 vaccination. However, the patient was discharged from the hospital with a satisfactory recovery after 16 days. The relationship between MIS and COVID-19 vaccines is not fully understood, and controlled studies are urgently required to reach a conclusion.

Importantly, COVID-19 infection may also be associated with the occurrence of MIS in children. Ooms et al. identified 18 cases of MIS among children out of 35,200 reported infections with an overall incidence rate of 7.2 per one million person-months. Fortunately, the prognosis for each of these children was excellent, and no deaths were reported. Martnez-Garca et al. also reported two cases of MIS after COVID-19 and suggested early identification of these cases for better prognosis and timely management. Hsu et al. reported a case of fulminant myocarditis that resulted in cardiogenic shock in a 9-month-old male infant. Nonetheless, the patient exhibited a favorable recovery with no significant morbidity. These findings demonstrate that MIS is uncommon in children who have been vaccinated, and the prognosis is significantly better than for COVID-19 infection itself.

The prevalence of COVID-19 infection is lower in children than adults and is primarily linked to their contact with infected adults (8). In addition, the severity of the disease is typically mild in children, but long COVID was still observed after infection recovery (9). Lokanuwatsatien et al. evaluated the prevalence of long COVID among children and found it to be 30.2%. Long COVID symptoms included appetite loss, rhinorrhea, and nasal congestion in children younger than three years old, and hair loss, dyspnea on exertion, rhinorrhea, and nasal congestion in children aged 3–18 years old. However, the prognosis was favorable among these children, but the follow-up of children with long COVID will still be of paramount importance. However, the relationship between vaccination and the occurrence of long COVID remains unclear and calls for further study.

The dissemination of information regarding the efficacy of COVID-19 vaccines to parents will unquestionably increase their confidence and subsequently decrease their reluctance to vaccinate their children. Chaiyakulsil et al. investigated the vaccination effectiveness in children, adolescents, and young adults during the Omicron era in Thailand. They found vaccinated individuals were less likely to develop infections than unvaccinated individuals. However, the vaccination’s efficacy is highly dependent on the number of doses administered. The authors have demonstrated that those who received three or four doses of any vaccine were more protected against infection than those who received only one or two doses. Similarly, the adjusted vaccination effectiveness for preventing at least moderate disease severity was greater among those who received three or four vaccine doses than among those who received one or two, regardless of vaccination schedule. Existing data indicate that side effects of COVID-19 vaccines among children are mild and transient, corroborating clinical trial reports. We believe that informing parents about the safety and efficacy of vaccinations will increase the proportion of vaccinated children.

Vaccine inequity has also been identified as a significant factor contributing to the poor performance of vaccination campaigns in numerous regions, particularly low and middle-income countries. Disparities in COVID-19 vaccination coverage among children and adolescents continue to exist (10, 11). To optimize vaccination coverage, the vaccine inequity in the pediatric population must also be addressed as a top priority. Bruckhaus et al. identified insufficiencies in vaccine uptake among certain pediatric populations in California and called for prompt resolution of these inequities and their resources, especially among vulnerable populations.

Collectively, these studies demonstrate that the childhood COVID-19 vaccination program is impacted by parental VH, which is primarily associated with concerns about vaccine safety and efficacy. Several socio-demographic characteristics of parents were discovered to be associated with vaccine reluctance. These findings have significant implications for healthcare administrators, professionals, and researchers. Health authorities should synthesize the research-based evidence and use it to design and implement targeted measures to increase the vaccination rate among children.
